# Selective Breeding for Genetic Improvement of *Nile tilapia* (*Oreochromis niloticus* Linnaeus, 1758) in Uganda: Current Status, Challenges, and Future Perspectives

**DOI:** 10.3390/ani15020142

**Published:** 2025-01-09

**Authors:** Ivan Abaho, Gerald Kwikiriza, Faith Atukwatse, Andrew A. Izaara, Joseph Ekwangu, Sylvester D. Baguma, Jerome Kubiriba, Nasser Kasozi

**Affiliations:** 1 Bulindi Zonal Agricultural Research and Development Institute, National Agricultural Research Organization (NARO), Hoima P.O. Box 101, Uganda; jekwangu@gmail.com (J.E.); sylvester.baguma@naro.go.ug (S.D.B.); 2 Institute of Integrative Nature Conservation Research, Department of Integrative Biology and Biodiversity Research (DIBB), University of Natural Resources and Life Sciences, Gregor Mendel Str., 33, 1080 Vienna, Austria; gerald.kwikiriza@boku.ac.at; 3 Department of Zoology, Entomology and Fisheries Sciences, College of Natural Sciences, Makerere University, Kampala P.O. Box 7062, Uganda; faith.atukwatse@mak.ac.ug; 4 Kachwekano Zonal Agricultural Research and Development Institute, National Agricultural Research Organization (NARO), Kabale P.O. Box 421, Uganda; jerome.kubiriba@naro.go.ug; 5 Mukono Zonal Agricultural Research and Development Institute, National Agricultural Research Organization (NARO), Mukono P.O. Box 164, Uganda; andrew.arinaitwe@naro.go.ug; 6 Buginyanya Zonal Agricultural Research and Development Institute, National Agricultural Research Organization (NARO), Mbale P.O. Box 1356, Uganda; nasser.kasozi@naro.go.ug

**Keywords:** aquaculture, breeding program, fish seed, genetic improvement, GIFT, growth rate, inbreeding

## Abstract

**Simple Summary:**

Aquaculture production in Uganda is dominated by *Nile tilapia*. With the recent growth of commercial cage aquaculture, *Nile tilapia* currently contributes up to 70% of the total farmed fish in Uganda. It is, therefore, a major source of animal protein and livelihood for at least 30% of the population. Although *Nile tilapia* is endemic to Uganda, it attains slower growth rates than the improved strains in other countries. The slow growth rate results in longer feeding periods, higher feed conversion ratios (FCRs), slow turnover, longer payback periods, and a depressed rate of return. Due to poor growth rates, a Ugandan farmer can only achieve one production cycle of 9 and 11 months in cages and ponds, respectively, compared to two cycles with the improved tilapia strains. This increases the cost of production, reduces profitability, and renders Ugandan tilapia farming less competitive compared with other producing countries using improved strains. As such, there is a need to establish a systematic selective breeding program focusing on strain improvement to guarantee the supply of fast-growing quality seed. This will contribute to the sustainable growth of *Nile tilapia* aquaculture in Uganda.

**Abstract:**

*Nile tilapia* (*Oreochromis niloticus*) aquaculture continues to significantly contribute to the growth of the aquaculture sector in Uganda. However, its production is beset by erratic and unreliable seed supply. Also, most hatcheries practice inbreeding of broodstock, resulting in inferior seed characterized by low growth rates. As such, a selective breeding program is necessary to readily avail fast-growing seed that respond to farmers’ needs. The present review consolidates available information on developing a *Nile tilapia* breeding program in Uganda. The article discusses the significance of genetic improvement, drawing lessons from successful *Nile tilapia* selective breeding programs in other countries. From a literature review, no systematic *Nile tilapia* selective breeding program was traceable in Uganda. Scanty information on the selective breeding efforts of the species was available, with little evidence of selection for improved performance. Overall, the national capacity for aquaculture research and development, including fish breeding and strain improvement, was weak and poorly funded. The review recommends purposive support for developing a systematic strain improvement breeding program, which will be a source of improved broodstock and seed for hatcheries and farmers, respectively. The program would guide the formulation of standard operating procedures for quality seed production towards ensuring sustainable aquaculture growth in Uganda.

## 1. Introduction

**Figure 1:**
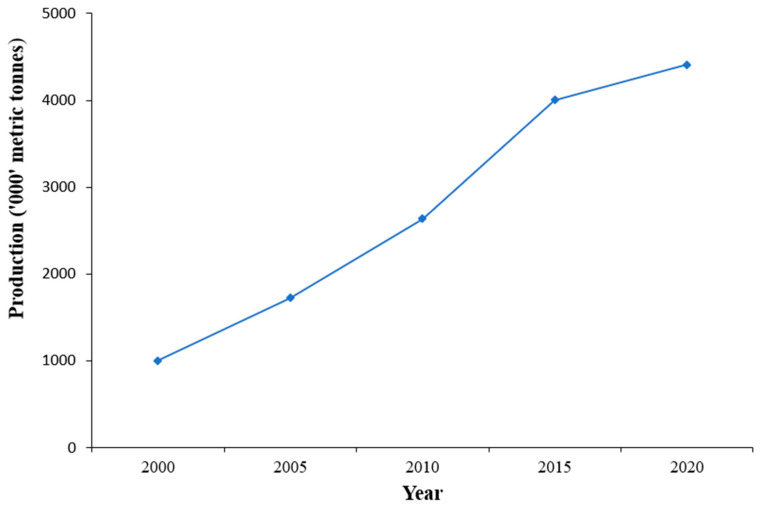
Global *Nile tilapia* aquaculture production from 2002 to 2020 [1].

Globally, aquaculture is regarded as one of the fastest-growing food-producing sub-sectors. For instance, in 2020, aquaculture contributed up to 49.2% of the 178 million tonnes of total aquatic animals produced globally [1]. Specifically, Asia has continued to dominate aquaculture production worldwide, with a contribution of 88.43% of the global production of aquatic animals in 2020, followed by the Americas (5.00%), Europe (3.74%), Africa (2.57%), and finally Oceania (0.26%) [1]. Although there are disparities in the level of production among the continents, the aquaculture industry has generally continued to grow faster than other food production sectors [2]. Therefore, aquaculture presents a viable option to meet the gap between the demand and supply of fish products arising from the stagnation of capture fishery production [3,4]. *Nile tilapia* (*Oreochromis niloticus*) is one of the major farmed fish species that contribute significantly to global aquaculture production. In 2020, it was ranked the third largest farmed finfish in inland aquaculture, after Grass carp (*Ctenopharyngodon idellus*) and Silver carp (*Hypophthalmichthys molitrix*) [1]. The fish is endemic to Africa, although it has been introduced into many countries globally, mainly for aquaculture [5,6,7,8,9]. Currently, *Nile tilapia* is grown in over 80 countries, and its global production has continued to grow, rising from 1.00 million tonnes in 2000 to 4.41 million tonnes in 2020 (Figure 1). As such, this fish species contributes significantly to global livelihoods as one of the primary sources of income and proteins for human consumption [1,4,10,11,12].

The rise in *Nile tilapia* aquaculture is attributed to the ease of breeding in captivity, the fast growth rate, the acceptability of artificial feeds after yolk-sac absorption, tolerance to a wide range of environmental conditions, and marketability [13,14]. Also, the high genetic diversity in the natural germplasm and adoption of all-male populations in culture systems, coupled with the development and dissemination of fast-growing strains through selective breeding, has contributed to the expansion of *Nile tilapia*’s global production [7,15,16,17,18]. For instance, the Genetically Improved Farmed Tilapia (GIFT) strain, developed through selective breeding of full sibling families and the selection of best-performing families as parents of the next generation, grows 30%–65% faster than non-improved strains and has been developed and widely adopted in Asia [19,20,21,22]. As such, Asian countries, i.e., China, Indonesia, Bangladesh, Thailand, and the Philippines, remain the major producers of *Nile tilapia* globally [1,6,7,10]. However, Egypt, from the African continent, is the third largest producer globally, only after China and Indonesia [6]. Egypt dominates the production of the species in Africa and contributed 84% (1,081,202 metric tons [MT]) of farmed *Nile tilapia* in 2019, distantly followed by Uganda (71,287 MT) and Ghana (45,760 MT) as the second and third highest producers, respectively [4,6,7]. Like in Asia, the development and adoption of the Genetically Improved Abbassa *Nile tilapia* (GIANT) strain has fueled aquaculture development in Egypt. As such, Egypt significantly contributes to global aquaculture output compared to other African countries [7,16,22,23]. Notably, the GIANT breeding program was initiated in 2002 by WorldFish Center, using local broodstock sourced from the river Nile. The program adopted GIFT technology to selectively improve the growth performance of the local strains. The resultant GIANT strain grew faster (28% faster growth rate), with a lower feed conversion ratio (15.7%) and a higher survival rate than the other commercial strains [24,25,26]. Overall, the improved *Nile tilapia* strains are estimated to contribute to more than 50% of the globally produced tilapia [27,28,29].

While Egypt significantly contributes to global *Nile tilapia* production, the production from other African countries has remained generally low, although with great potential due to the existence of a significant wealth of tilapia genetic resources [1,30]. This is attributed to the weak and poorly funded aquaculture research industry, with inadequate or no investment in genetic improvement, focusing on developing fast-growing strains. Besides, there is low adoption of already existing genetically improved strains in many African countries, driven by concerns that exotic strains will contaminate the native strains, consequently compromising their conservation [4,22,30]. Therefore, poor quality fish seed, with inferior growth performance, remains one of the major barriers to the development of aquaculture in Africa [22,30,31]. In Uganda, the supply of quality *Nile tilapia* seed is erratic and unreliable, with farmers unable to readily access the quality and quantity needed, hence limiting aquaculture development [4,32,33]. Moreover, the available seed attains slower growth rates than the improved strains [34]. Nonetheless, some interventions for *Nile tilapia* strain improvement focusing on strain improvement have been initiated in Uganda. Therefore, the present review article consolidates existing information on enhancing *Nile tilapia* aquaculture in Uganda, focusing on genetic improvement through selective breeding to produce superior seed and broodstock. The article draws on research carried out by the Ministry of Agriculture, Animal Industry, and Fisheries (MAAIF), the National Agricultural Research Organization (NARO), the National Animal Genetic Resources Centre and Data Bank (NAGRC&DB), and farmers to provide information on the current state of development of a selective breeding program for *Nile tilapia*. The significance of genetic improvement through selective breeding of the species, with reference to experiences from other regions, is also presented. Finally, the review presents future perspectives for developing a systematic breeding program in Uganda.

The literature search strategy involved desk reviews probing for information from Web of Science, Science Direct, Google Scholar, and Scopus using key search terms “Breeding programs”, “Selective breeding”, “Tilapia”, and “*Nile tilapia*” as keywords. Besides the primary publications that fulfilled the search criteria, more articles were obtained by scanning the relevant cross-references. Conference proceedings, academic theses, and reports from the private sector and government entities, mainly MAAIF’s Directorate of Fisheries Resources (DiFR) and their agencies, specifically NARO’s Zonal Agricultural Research and Development institutes (ZARDIs) and the National Fisheries Resources Research Institute (NaFIRRI)-Kajjansi, were utilized. The search results were screened and selected based on title relevance to the present review regarding the selective breeding and genetic improvement of *Nile tilapia* prior to inclusion in the article.

## 2. *Nile tilapia* Aquaculture in Uganda

**Figure 2:**
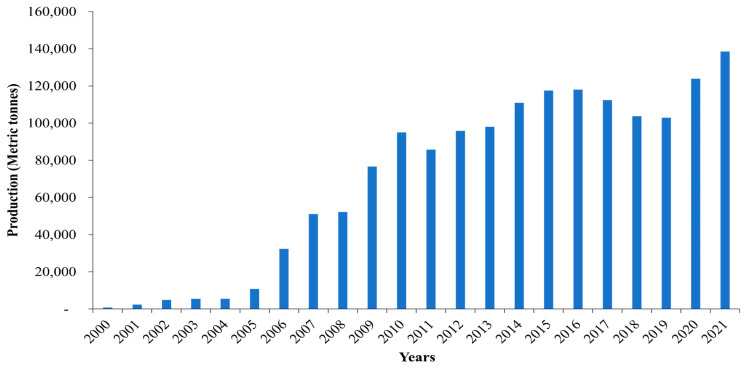
Aquaculture production in Uganda from 2000 to 2021 [37].

**Figure 3:**
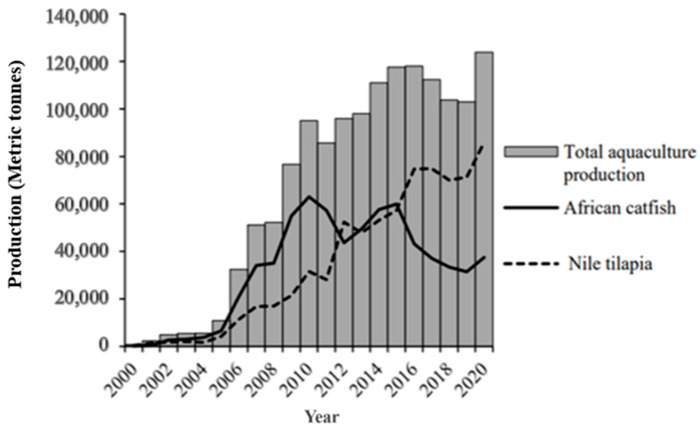
The trend in the production of major farmed fish species in Uganda [53].

Fish remains one of the significant sources of animal protein and livelihood for at least 30% of the population in Uganda [35,36]. For example, the per capita fish consumption in 2019 (about 13.3 kg) was derived from 470,000 MT of capture fisheries harvest and 102,943 MT of production from aquaculture [37]. However, with declining capture fisheries, aquaculture is seen as one of the feasible solutions for increasing the benefits derived from fish. As such, in their National Fisheries and Aquaculture Policy (NFAP), Uganda aims to produce at least 1,000,000 metric tons (MT) of farmed fish per annum by 2030. Uganda is endowed with fresh water, including the five major lakes (Victoria, Kyoga, Albert, Edward, and George), rivers, valley dams, and ponds, whose potential, if maximally harnessed, can significantly contribute to the NFAP target [37,38]. The continued investment in aquaculture has already yielded a considerable increase in farmed fish production, from 820 MT in 2000 to 138,558 MT in 2021 [37] (Figure 2). Two major fish species, i.e., *Nile tilapia* and the African catfish (*Clarias gariepinus*), continue to dominate the aquaculture industry, with the former species as the most farmed fish [35] (Figure 3), estimated to contribute up to 70% of the total aquaculture output in Uganda. The increase in farmed *Nile tilapia* production is attributed to the recent significant investments in cage aquaculture, with over 3000 cages operating on various Ugandan water bodies, all rearing this species [39].

Even though aquaculture production has tremendously increased in Uganda, the sector is still constrained by limited investment in fish farming, a high rate of abandoning fish farms, limited access to quality fish seed and feed, and inadequate extension services coupled with inadequate human, technological, and infrastructural capacity leading to low production and productivity [32,36,40,41,42,43,44]. Specifically for *Nile tilapia*, its crucial role in Uganda’s aquaculture is still constrained by poor quality seed characterized by poor growth rates, which has had a knock-on effect on aquaculture production volumes [34,45,46,47]. For example, most hatcheries produce seed from mismanaged broodstock, leading to inbreeding and unviable, sometimes deformed offspring with low productivity. This is worsened by the weak enforcement of standards. Also, the existing formal seed system cannot supply quality seed to farmers in the required quantities and at the right time. Farmers, therefore, rely on the informal sector, getting seed from the wild or smallholder seed multipliers whose seed quality is highly compromised, mostly by mixed species and of an unknown genetic makeup [47]. The utilization of poor-quality seed continues to result in slower growth rates compared to the improved strains, resulting in more extended production cycles, higher feed conversion ratios (FCRs), slow turnover, longer payback periods, and a depressed rate of economic returns. Consequently, a Ugandan farmer can only achieve one production cycle of 8 and 12 months in cages and ponds, respectively, in a year, compared to two cycles with the GIFT and other improved strains [23,27,48,49,50]. This increases the cost of production, reduces profitability, and renders Uganda’s *Nile tilapia* fish farming less competitive in comparison with other producing countries such as Egypt, Ghana, China, and the Philippines [6,23,51,52].

To solve the challenge of poor *Nile tilapia* seed in Uganda, a systematic selective breeding program is needed to guarantee the supply of quality seed. A well-programmed selective breeding program allows for the isolation of desirable traits, including superior growth and survival rates [20,54,55,56]. The selective breeding approach is inadequately deployed in Uganda’s *Nile tilapia* aquaculture. Common practice involves randomly obtaining tilapia broodstock from the wild (lakes and rivers) to hatcheries, with no deliberate selection regimes and accurate strain separation, resulting in seed contamination with other tilapia strains, which lowers growth performance. Also, the fish population histories of Ugandan lakes are awash with multifarious fish introductions, which greatly complicate the identification of genetically productive broodstock for aquaculture [57]. As such, a properly designed selective breeding program is urgently needed to provide a solution to the inferior *Nile tilapia* seed and guarantee sustained aquaculture growth.

## 3. Selective Breeding in Aquaculture

Selective breeding involves harnessing the genetic variation in desirable traits within a population to improve the production quality and efficiency of the next generation of the target species [20,58,59,60,61]. In aquaculture, selective breeding is envisioned to result in genetic improvement where a selection of desirable traits in the farmed fish is conducted for genetic improvement and subsequently enhances commercial production [62,63]. As such, the process results in the following: (1) increased productivity; (2) improved economic value; and (3) improved utilization of resources, consequently increasing the production and sustainability of aquaculture [58,64,65,66]. For example, a significant increase in the production of *Nile tilapia* [67], carp, and salmon [68,69,70] has been reported due to genetic improvement. Notably, selective breeding in aquaculture is preferred for genetic improvement compared to hybridization, crossbreeding, chromosome manipulation, sex control, and transgenesis since it allows for continued genetic gain from generation to generation [71,72,73]. However, conventional selective breeding has some drawbacks, such as low efficiency and a long breeding cycle. With advances in genome sequencing, marker-assisted selection (MAS), and genomic selection (GS) that apply genomic information, limitations to traditional selective breeding have been disentangled [56,74,75,76,77]. These approaches help to precisely estimate the breeding values and accelerate the selection process for desired traits, shortening the production cycle.

The establishment of a selective breeding program is a systematic process comprising the assemblage of the required germplasm and establishment of a base population, the development of breeding objectives, and selection strategies [23,78,79,80]. A breeding objective is crucial because it determines “where to go” with the genetic improvement program. Also, the traits selected for the program should be of commercial importance in the actual production system, i.e., traits that will impact upon income or expense in the production system or those associated with benefits to the user of the improved fish. As such, a sustainable breeding program must take into consideration the consumer preferences and the aspirations of other stakeholders, including the government. Further, the current and future demand of the species targeted for genetic improvement must be analyzed before the initiation of the genetic improvement program. Consideration for the required funding and the respective funding mechanisms, the necessary human resource capabilities and research and production facilities, and the required policy direction is vital [23]

**Figure 4:**
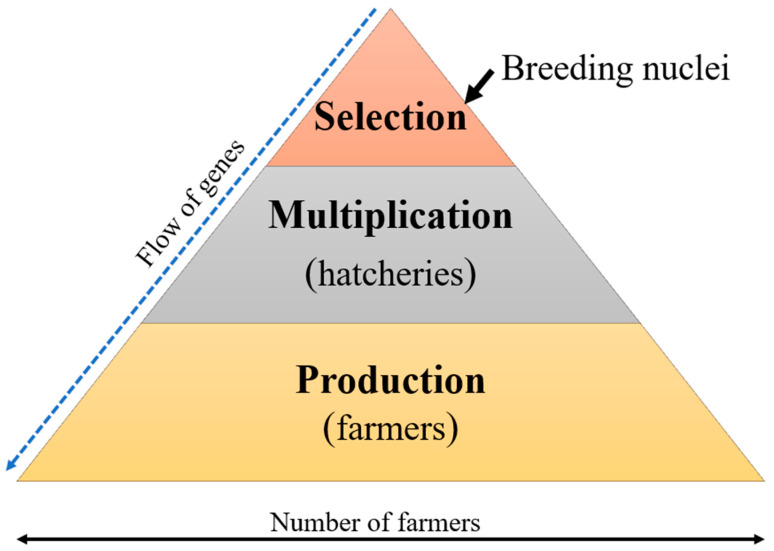
Dissemination approach for improved *Nile tilapia*.

The breeding objective may include traits such as growth rate, survival rate, disease resistance, tolerance to water temperature, and flesh quality [62,65,81,82]. Notably, the growth rate remains the popular trait in most breeding programs in aquaculture because of its paramount importance in the production system [58,62,82,83]. In a production cycle of fixed duration, a higher growth rate results in larger fish with a shorter production period, increased productivity, and, subsequently, shorter payback periods. During the growth rate improvement program, the first-generation best-performing fish individuals are selected to produce the progeny for the second generation [84,85]. The benchmark is that the resulting population is genetically improved with a higher phenotypic mean of the desired traits compared to the founder population. Usually, genetic improvement occurs in a very small proportion of the population, with the upgrade achieved in the “elite” individuals, who are multiplied and disseminated to the production systems (Figure 4).

While establishing a breeding program, the quality of the initially used broodstock to produce seed per production cycle is important. Notably, a sufficient number of broodstock contributing to the next generation, i.e., at least 50 pairs of breeders as the effective breeding size (Nb), helps reduce risks of inbreeding and its associated effects on the offspring [85,86,87,88]. Replacement of brood fish is vital due to the declining vigor with time and brood fish mortality, and is also key in maintaining genetic diversity. Also, the starting size for the broodstock is critical; for example, in *Nile tilapia*, fish ranging from 150 to 250 g are appropriate since they can produce 500–1000 eggs per spawning. Notably, the number of eggs decreases with the age and size of broodstock, hence requiring broodstock replacement after 24 months [47,89,90].

### Selective Breeding of Nile tilapia

In *Nile tilapia* aquaculture, producing heavier fish in a shorter period of the preferred flesh quality means more income for farmers and significant economic benefits. Selective breeding can be utilized to achieve these attributes since it results in long-term genetic gain and trait improvement [62,71,72,91]. With systematic selection, quality seed is produced and availed to farmers, which subsequently results in sustainable aquaculture development. In *Nile tilapia* aquaculture, selective breeding started in the early 1990s and has, over time, significantly contributed to making the species one of the most important farmed fish globally. Numerous selective breeding programs of *Nile tilapia* have been established (Table 1), with the GIFT strain as the major success story [23,48,55,68,92,93]. With these breeding programs, remarkable genetic improvement has been achieved over the years [23,48,55,58,67,68,92]. The programs mostly utilize the classical selection approach using mass spawning or pedigree information, with growth as the main trait of interest [21,23,24,54,55,84,91]. However, marker-assisted selection is gaining momentum in routine selection programs as a more efficient and effective breeding approach compared to conventional techniques [56,76].

Notably, the selective programs in *Nile tilapia* aquaculture have mainly been implemented and disseminated among Asian countries, resulting in increased aquaculture growth. For example, the GIFT program was initiated in the Philippines, and the resultant improved strains have been adopted mostly in Southeast Asia, resulting in improved productivity ranging from 18% to 58% in China and Bangladesh, with each generation yielding 7–10% gains in productivity [6,22,48,68,92,94]. Consequently, Asia remains the leading producer of the species [1,6,7,23,67]. In African countries, the national capacity for aquaculture research and development, including fish breeding and strain improvement, is still weak, with inadequate technical human resources and limited funding [22,23]. Subsequently, the farmed fish strains continue to yield sub-optimally compared with improved strains in other regions. The notable breeding programs in Africa are for the Genetically Improved Abbassa *Nile tilapia* (GIANT) strain and the Akasombo strain in Egypt and Ghana, respectively. The Abbassa and Akosombo strains reach their harvest weight faster compared to non-improved strains, saving both time and money for farmers in terms of labor and fish feed costs [23]. The GIANT strain is responsible for the trajectory growth of aquaculture in Egypt, is presently the leading producer of *Nile tilapia* in Africa, and is among the top ten global producers, justifying the need for systematic selective breeding programs in the continent [1,6,25,26]. The other breeding programs in Africa are utilizing the GIANT selective breeding protocol to produce fast-growing strains, especially in Malawi, Zambia, Ghana, and Kenya [20,23,95]. However, the distribution of genetically improved strains for the purpose of aquaculture in Africa is still limited by the concerns of contaminating native genetic diversity [21,23].

**Table 1:** *Nile tilapia* improved strains developed through selective breeding programs for different countries.

Country	Strain	Institution	Year Started	Germplasm Source	Target Trait (s)	Generation	Benefits	Reference
Philippines	GIFT	Bureau of Fisheries and Aquatic Resources and Freshwater Aquaculture Centre (BFAR) of Central Luzon State University	1988	Four wild strains from Africa (Egypt, Ghana, Kenya, and Senegal) and four farmed strains in the Philippines	Growth and survival rates	6th	The 6th generation had 77% faster growth and 60% higher survival rates than locally farmed strains in the Philippines. An average genetic gain of 12–17% per generation across five generations of selection was obtained	[8,65,93,96,97,98,99,100]
	FaST or FAC-Selected Tilapia	Freshwater Aquaculture Center Central Luzon State University (FAC-CLSU)	1986	*Nile tilapia* strains were collected from Taiwan, Singapore, Thailand, and Israel. These were referred to as the Philippines strain	Growth rate	12th	A genetic gain in body weight of 12% per generation was observed after 12 generations of selection	[13,97,101,102]
	GET EXCEL	National Freshwater Fisheries Technology Center, Bureau of Fisheries and Aquatic Resources	2002	Strain crosses and within-family selection using four parent lines: 8th generation GIFT, 13th generation FaST, and *Nile tilapia* from Egypt and Kenya	Disease resistance, growth, and survival rates	1st	The strain was more disease resistant, with higher growth and survival rates compared to the 8th generation of GIFT	[13,97,103,104]
	GIFT	TGA Farm Incorporated	2006	GIFT from WorldFish Center	Growth rate	2nd	Fast growth performance, coupled with increased farm revenues	[68,92]
Malaysia	GIFT	The WorldFish Center	2001	6th generation from the GIFT project in the Philippines	Growth rate	10th	An accumulated response of 107% in growth rate, averaging 11.9% per generation	[96]
China	NEW GIFT	Shanghai Ocean University	1994	3rd generation of the GIFT project in the Philippines	Growth rate	8th	Higher growth rate (>30%) than the base population	[105,106,107,108]
	GIFT	Freshwater Fisheries Research Center (FFRC), Chinese Academy of Fishery Sciences (CAFS)	2006	The GIFT project in the Philippines	Growth rate	3rd	Superior growth performance than that of the existing strains	[106,109]
	Hainan Progift	Hainan Progift Aqua-Tech Cooperation Ltd.	2005	5th generation of *Nile tilapia* from the Vietnam National Breeding Program, earlier obtained from the GIFT of the Philippines	Growth rate	6th	Genetic growth improvement (60–90%) larger body weight at harvest) after six generations of multi-trait selection	[63]
Egypt	GIANT	The WorldFish Center, Egypt	2002	Sourced from the river Nile	Growth and survival rates	9th	The generation of the GIANT strain grew 28% faster than the commercial strain	[24,25,68,110]
Ghana	Akosombo strain	Water Research Institute (WRI)	2002	11th generation of the GIFT strain imported from WorldFish Center in Malaysia	Growth and survival rates	10th	The 10-year selection resulted in the Aksombo strain with a 30% faster growth rate than other farmed tilapia strains in the West African sub-region	[68,95,111]
Kenya	Sagana strain (SAG-F8)	National Aquaculture Research Development and Training Centre, Sagana, Kenya	2010	Private and government hatcheries and lakes Victoria and Turkana	Growth and survival rates and FCR	8th	Faster growth rates, improved feed efficiency, and better survival rates	[20,51]
Norway	GenoMar Supreme (GST) Strain	GenoMar, a Norwegian company based in Oslo	1999	10th GIFT generation	Growth and survival rates	10th–13th	Increased genetic gain (20% per generation). An increase in the survival rate of about 11% per generation was also observed, resulting in the survival of at least 80% in the 13th generation	[13,21,97,103]
Bangladesh	GIFT	Bangladesh Fisheries Research Institute (BFRI)	1998	The GIFT strain from the Philippines	Growth rate	6th	F6 generation progeny showed 32.66% higher growth than that of the average group of the GIFT strain (base population)	[112]
Vietnam	NOVIT 4	Research Institute for Aquaculture No. 1 (RIA 1)	1997	5th generation of the GIFT from the Philippines and Thailand tilapia	Growth rate	8th	Growth improvement, well suited to production systems, with a 52% superior growth rate than the base population	[68,92,113]
	GIFT	Research Institute for Aquaculture No. 2 (RIA 2)	2006	10th generation of the GIFT strain developed in the Philippines	Harvest weight	4th–6th	Genetic gains in harvest weight, ranging between 7 and 11% per generation over four to six generations	[67,68]
Sri Lanka	GIFT	National Aquaculture Development Authority (NAQDA) of Sri Lanka	2007	GIFT from Malaysia	Growth and survival rates	4th	Achieved 112% greater growth than the local strain and an 85.4% survival rate compared to 75.5% for the local strain	[68,114,115]
Brazil	GIFT	State University of Maringá, Brazil	2005	8th generation of GIFT from Malaysia	Growth rate	1st–9th	A substantial increase in the daily weight gain of about 3.3% per generation	[116,117]
	Aquabel strain	Aquabel Pisciculture Station	1996	The Chitralada strain from the Asian Institute of Technology (AIT)	Growth and survival rates	N/S	Fast-growing strain with the higher survival rate (94.1%)	[118,119]
	AquaAmerica strain	AquaAmerica Company, Brazil	2012	GIFT previously crossed with the Chitralada and Bouaké strains	Growth rate	3rd	Superior growth rates exhibited by the strain	[120]
Thailand	Big Nin	Nam Sai Farms Co. Ltd.	N/S	The GIFT strain from the Philippines	Growth rate	N/S	* A fast growth rate of 2.5 g/day in ponds and 4.4 g/day in cages	[121]
	GIFT	Manit Farm	2008	Thai stocks and from GIFT	Growth and survival rates	9th	Enhanced harvest weight and survival rate	[68,91]
		Pathum Thani Fisheries Test and Research Center (Pathum Thani FTRC	2000	9th generation GIFT from the WorldFish Center, Malaysia	Growth rate	5th	Superior growth performance	[68,122]

GET EXCEL: genetically enhanced tilapia—an excellent strain; GIANT: Genetically Improved Abassa *Nile tilapia*; GIFT: Genetically Improved Farmed Tilapia; FCR: feed conversion ratio; N/S: not specified; *: https://www.tilapiathai.com/tilapia-strains/ (accessed on 19 April 2024).

## 4. Existing *Nile tilapia* Genetic Resources, Seed Production, and Genetic Improvement Efforts in Uganda

### 4.1. Existing Nile tilapia Genetic Resources in Uganda

**Figure 5:**
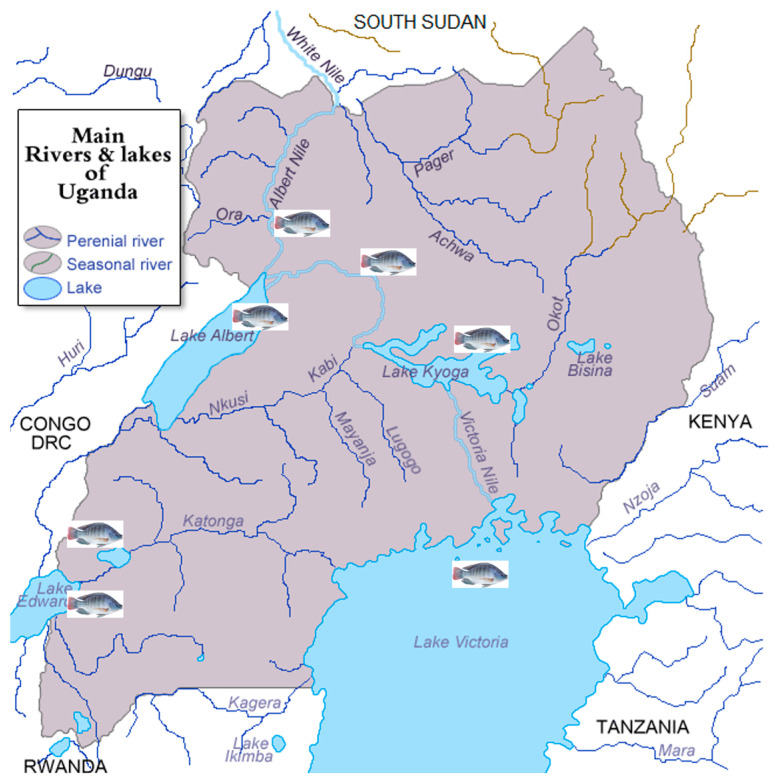
Major water bodies (lakes and river systems) in Uganda where *Nile tilapia* is naturally distributed.

While establishing a breeding program, the existing genetic resources are indispensable building blocks. Uganda is endowed with vast *Nile tilapia* genetic resources across various major water bodies (Figure 5), small water systems, and fish farms, which are potential broodstock sources in a breeding program. Notably, the species is native to lakes Edward, George, and Albert but was introduced to the Lake Victoria basin (lakes Victoria, Kyoga, Nabugabo, and the Victoria Nile) as well as various satellite lakes [6,8,46,123]. However, the diverse *Nile tilapia* genetic resources in Uganda have not yet been fully explored and utilized in aquaculture. Although some efforts for the genetic improvement of *Nile tilapia* are ongoing, the question is how the wealth of wild resources shall be sustainably used. Besides, with the increase in cage fish farming investments in major Ugandan water bodies, cases of intentional or unintentional fish escapees are inevitable and may pose threats to the native species. The intentional release of fish, especially of unknown origin, or the accidental escape of fish from farms have been linked with hybridization with native species and admixture between stocks [124,125,126]. Hybridization can result in reduced genetic adaptation arising from the break-up of co-adapted gene complexes, which is detrimental to the sustainability of genetic resources [95,126,127]. Overall, the escapes from fish farms interbreed with the native wild fish populations, compromising their population structure, including reduced genetic diversity and fitness [95]. Consequently, the farmers collect broodstock which is not pure stock, having poor performance on the farms. Nonetheless, the genetic diversity of the Ugandan wild *Nile tilapia* populations is generally high, with genetically healthy breeding populations. The wild stock remains the main source of broodstock for seed producers, with lakes Albert, Kyoga, Edward, and George, as well as the river Nile and the Kazinga channel, having a significant genetic contribution to most farmed *Nile tilapia* populations in Uganda [128,129].

### 4.2. Nile tilapia Seed Production in Uganda

In Uganda, there are several isolated *Nile tilapia* hatcheries, with the majority located in the central and eastern regions of the country [47] (Table 2). Seed production is mainly private sector-led, with small-scale production in ponds of 100–500 m^2^ and an annual capacity of 200,000 to 300,000 fingerlings [31,47,130]. Farmers and hatchery operators use rudimentary fish seed production technologies and hence cannot guarantee quality seed. For example, a significant number of hatcheries source their parental stock from the already genetically degenerated broodstock of other farms, which is evidenced by the weak genetic differentiation between the *Nile tilapia* hatchery populations, which can be attributed to genetic mixing [129]. Notably, a few hatchery operators get their germplasm from natural water bodies such as lakes Victoria, Kyoga, Albert, George, and Edward but never screen for their genetic makeup [47,128]. The cost of the breeders generally influences the sourcing of broodstock by the fish hatcheries, in addition to the proximity of the source and the availability of broodstock. Therefore, the hatchery operators do not follow quality considerations like ancestry and performance for broodstock during sourcing. Such practices, coupled with unregulated fish translocations and the prolific breeding of *Nile tilapia*, result in a rapid reduction in the genetic quality of the farmed fish due to inbreeding and genetic drift. Consequently, genetic variability is lost, resulting in poor-quality populations with low growth and survival rates and, therefore, low yields [61].

Also, with a few exceptions, small-scale seed producers commonly use the single-pond fingerling production system, where fish fingerlings are produced and nursed in one pond with broodstock [47]. In this system, fingerlings’ production cycles overlap, causing them to reach sexual maturity and consequently resulting in inbreeding. This leads to a loss of genetic variability and, hence, poor-quality *Nile tilapia* seed, characterized by low growth and survival rates [31,131]. Subsequently, the hatchery operators supply inferior seed to farmers, resulting in low yields and depressed economic returns from the investments.

Given the above situation, establishing a systematic breeding program focused on the genetic improvement of the species is vital for the country. Selective breeding has resulted in genetically improved tilapia, which has been proven to have faster growth rates, better survival rates, and better disease resistance [13,20,25,26,27,34,111]. With classical selection techniques for growth traits, genetic gains in body weight of *Nile tilapia* by 20–90% have been reported [21,34,54,55,56,63,84,91]. However, the aquaculture industry is now moving to the use of robust molecular-based marker-assisted selection (MAS), and breeders can now indirectly choose genetic traits of interest using linked markers. This approach hastens the selection for fast growth in *Nile tilapia*, shortening the production cycle [56,74,75,76,132]. Since aquaculture research and development is still at an early stage in Uganda, with no systematic breeding efforts of *Nile tilapia*, the country can benefit from the already-generated knowledge utilizing the two approaches.

### 4.3. Genetic Improvement Efforts of Nile tilapia in Uganda

With respect to research towards creating a *Nile tilapia* selective breeding program in Uganda, most studies have been conducted to establish the genetic diversity of the existing wild resources. Notably, most of these efforts have been implemented using donor-funded projects, such as the Strengthening Regional Capacity in Research and Training in Fisheries and Aquaculture for Improved Food Security and Livelihoods in Eastern Africa (STRECA-FISH) project, the European Union’s Promotion of Environmentally Sustainable Commercial Aquaculture (PESCA) project, and the GCRF AgriFood Africa Innovation Awards round three grant via Innovate UK KTN [128,129]. From these projects, three discrete *Nile tilapia* population structures were established: (1) lakes George and Edward, as well as the Kazinga channel; (2) lake Albert, the river Nile, and Kyoga; and (3) the Lake Victoria systems [128,133]. Genetic distance analysis demonstrated a closer genetic relationship between fish from lakes Kyoga and Albert, while the fish from Lake Victoria were genetically distinct from those of the other two lakes [128,129]. Also, admixture analysis studies showed that the Lake Victoria population was predominantly pure stock, while the lake Kyoga stock was admixed, sharing significant genetic composition with the pure stockfish from lake Albert [129]. Taken together, the information from these studies will support efforts to develop a selective breeding program, especially by providing credible sources for germplasm and continuous monitoring of inbreeding and genetic diversity levels of the selected populations for the breeding program.

Also, through the World Bank-funded “Agricultural Technology and Agribusiness Advisory Services” (ATAAS) project, researchers in Uganda attempted to develop a fast-growing *Nile tilapia* strain through selective breeding [134]. The NARO team sourced the initial population comprising farmed strains from the Aquaculture Research and Development Center (ARDC)-Kajjansi hatchery and wild populations from lakes Albert, Edward, Kyoga, and Victoria. The trial was performed at ARDC-Kajjansi, aiming to develop a strain that would attain higher harvest weight within a shorter time. The results indicated that the Lake Victoria strain had a better daily growth rate of 2.47 g per day, which was a significant improvement from the previously reported rate of 0.52 g per day [34,134]. However, there were no comprehensive studies from the controlled culture conditions to field farms to determine the growth rates arising from genotype by environment interaction. This would confirm whether the reported genetic gain would not be lost when selection is not undertaken to address different categories of environments [135,136].

The above studies provide salient baseline information to the selective breeding research group and hatchery operators. However, the results are still insufficient to guide robust decisions in line with the development of a systematic *Nile tilapia* selective breeding program in Uganda.

## 5. Policy and Legal Framework for *Nile tilapia* Breeding in Uganda

The management and development of sustainable commercial and small-scale aquaculture in Uganda is governed by the MAAIF through the DiFR. The directorate provides technical guidance on the formulation, review, and implementation of policies, legislation, standards, plans, and strategies in fish production, marketing, inspection, and certification [137]. While supporting and promoting aquaculture production and management, the operations of DiFR are guided by the Fisheries and Aquaculture Act (2022), the Fish (Aquaculture) Rules (2022, 2003) and the Ugandan NFAP (2017). Under Fish (Aquaculture) Rules 2022 statutory instrument number 97, any person, institution, organization, or establishment intending to engage in fish breeding must have a fish breeding permit [38,138,139,140]. The permit may be given or withheld depending on the requirements of the fish breeding code and practices set forth by the DiFR. Also, under the same rules, all fish seed-producing persons or establishments must be certified to ensure that they produce quality fish seed following guidelines and the code of aquaculture practices. Before undertaking fish breeding operations, the farms should be assessed so that all activities align with policies. However, due to limited resources, most hatcheries are not licensed, with the farms lacking knowledge of policy rules and guidelines related to hatchery establishment and management [47]. As such, there is a significant unregistered supply of seed and inadequate quality checks for the few registered supplies.

In addition, NARO, an agency under MAAIF, is mandated to oversee and guide all aspects of agricultural research in Uganda, including fisheries and aquaculture. Through their ZARDIs and the ARDC-Kajjansi under the National Fisheries Resources Research Institute (NAFIRRI), NARO conducts research on fish breeding and genetic improvement. However, the Animal Breeding Act of 2001 established the NAGRC&DB, also an agency under the MAAIF, to establish sustainable national animal breeding programs, including fish breeding and genetic improvement [141]. As such, the mandates of the two agencies regarding fish breeding overlap, leading to the sharing of meager government resources meant for breeding and genetic improvement research, which would otherwise be entirely directed to establishing the country’s only fish breeding program. The two agencies implement their activities following the same policy documents regulating the aquaculture industry, including hatchery establishment and operations for various fish species comprising *Nile tilapia*.

Notably, the Uganda’s NFAP identifies inadequate access to quality fish seed as a significant challenge for aquaculture development in the country. The major issues highlighted by the policy include limited public and private investment in quality improvement and seed production, poor quality/counterfeit seed on the market, and ineffective coordination among relevant authorities in the research and regulation of fish seed. It, therefore, suggests the following strategies for overcoming the challenges related to limited access to quality seed: (1) supporting public-private partnerships to ensure effective seed production; (2) enhancing the capacity of NaFIRRI to execute its mandate effectively; (3) enhancing coordination efforts of the DiFR, NaFIRRI, NaGRIC, academia, and other research organizations, (4) strengthening the enforcement of existing laws and regulations to ensure compliance among critical actors in fish seed value chain; (5) supporting the private sector to produce quality seed using set standards by incentivizing regulation abiders and penalizing those who contravene the regulations; and (6) strengthening existing institutional systems for fish seed inspection and quality control.

## 6. Future Perspectives

Despite the significance of *Nile tilapia* genetic improvement programs, investment towards the conservation and selection of the *Nile tilapia* germplasm in Uganda has remained insufficient. The country has made little effort to establish a well-planned *Nile tilapia* selective breeding program for sustainable and cost-effective aquaculture production. This is coupled with the lack of standardized hatchery practices and the poor enforcement of policies to regulate and monitor the operations of hatcheries in the country, resulting in insufficient quality control for seed quality. Given the evidence of better performance by improved *Nile tilapia* obtained from local strains in Asia, Egypt, and Ghana [17,19,20,23,24,26,29,92,108,111], committing sufficient resources towards developing a systematic breeding program is critical.

Various stakeholders continue to push for the transfer/introduction of already existing improved strains into Uganda. This, however, is not encouraged to avoid the risk of potential adverse impacts on native germplasm, such as genetic integrity and the unknown effects of gene–environment interactions. Developing a local breeding program based on locally available strains of *Nile tilapia* in Uganda is highly recommended. This necessitates long-term significant commitment of human, infrastructural, and financial resources, including committing adequate resources to appropriate dissemination strategies such as the establishment of broodstock multiplication centers (BMCs), hatcheries, and a functional feedback mechanism. This requires competent staff with sufficient incentives, an appropriate succession plan, and the availability of functional breeding facilities. Uganda can, however, adapt the already developed selective breeding protocol in Asia, Egypt, or Ghana for use in the genetic improvement of *Nile tilapia* in Uganda, including testing other approaches for genetic improvement.

Although some government agencies and private commercial hatcheries are conducting *Nile tilapia* selective breeding activities [34,134], the interventions need to be coordinated so that a sustainable national breeding program is established. The funding from the government continues to dwindle, coupled with dependence on donor funding, which runs for a few years at a time. Without consistent funding, the establishment of the breeding programs and, later, the maintenance of the achieved genetic gains during the project’s lifetime further hinder efforts to develop and maintain a systematic breeding program [23,31,71]. Notably, the genetic improvement programs require an initial significant investment, as well as recurrent annual expenditure, to run them. Given the resources required for breeding programs, government institutions may remain unconvinced about investing in them unless they result in significant benefits [142]. Therefore, Uganda’s envisioned *Nile tilapia* breeding program should adapt to what other breeding programs have already done and also take advantage of advancements in genetics and breeding technologies to minimize the cost and realize the promise of providing a fast-growing strain. This would be a breakthrough in reducing the cost of production, contributing to a profitable aquaculture sector in Uganda.

Also, although the current efforts to develop a selective breeding program for *Nile tilapia* are based on developing fast-growing strains from local strains, targeting improving the weight at harvest, isolated and small selection experiments are still practiced from controlled environments at research stations. In most breeding programs, the realized responses in farm environments are always poor, with reports that genetic gains from selection carried out in good and environmentally controlled environments are often lost or reduced when the improved fish are exposed to less favorable farm conditions [143,144,145]. Therefore, as Uganda embarks on developing a sustainable breeding program for *Nile tilapia*, there is a need to incorporate well-planned studies in different culture conditions across all agro-ecological areas, including low-input production systems, where farmers grow *Nile tilapia* in ponds fertilized with organic material alone or feed the fish on a wide variety of locally available farm resources.

Notably, as the Ugandan aquaculture sector continues to grow, the sustainable use of *Nile tilapia* genetic resources requires the active involvement of multiple stakeholders. As such, the formation of a diverse national fish breeding and genomics research group to spearhead the formation of a national breeding program is vital. The group would undertake molecular genetic diversity studies, genetic improvement of commercial fish species, risk analysis, and recommend appropriate policy interventions. The research group would comprise aquaculture researchers, private commercial hatcheries, and policymakers. Also, the involvement of multinational organizations such as the Food and Agriculture Organization (FAO) and WorldFish Center to provide technical and financial assistance is vital.

**Figure 6:**
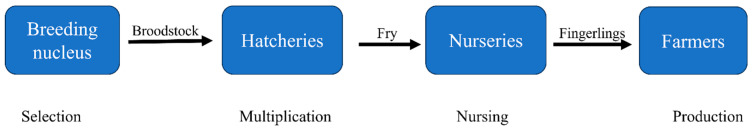
Proposed *Nile tilapia* breeding model for Uganda.

Establishing a sustainable breeding program requires having hatcheries as multiplication centers, which ideally receive a new generation of improved broodstock from the breeding nucleus every three years to sustain the production of improved seed. As such, this review proposes a simple selective breeding model for Uganda, as described below (Figure 6). The model proposes the government’s aquaculture research institutions as sources for breeding nuclei for producing improved seed and broodstock. The genetically improved seed is multiplied by certified private and government-owned hatcheries that obtain improved broodstock from the breeding nucleus, and finally, the seed is disseminated to farmers for grow-out production.

## 7. Conclusions Prioritized

This review showed that adopting improved strains has significantly contributed to an accelerated increase in the production of *Nile tilapia* in Asian countries and Egypt (Africa). Therefore, it is imperative that genetic improvement of local strains is prioritized to enable sustainable growth of *Nile tilapia* aquaculture in Africa. Similarly, considering the growing importance of *Nile tilapia* in Uganda, genetic improvement of the species must be given more attention with a focus on streamlining research interventions towards developing a well-planned *Nile tilapia* selective breeding program. This will guarantee the supply of superior seed, which will significantly contribute to increased returns on investments by fish farmers and, consequently, aquaculture growth in Uganda.

## Figures and Tables

**Table 2:** *Nile tilapia* hatcheries in Uganda.

District	Sub County/Division	Name	Ownership
Wakiso	Ssisa	Aquaculture Research and Development Centre-Kajjansi	Public
	Katabi	Tende Innovation Farm	Private
	Wakiso	Aquafarm Fish Farm	Private
	Katabi	Victoria Treasures	Private
	Mende	Kakunyu Agricultural Farm	Private
	Nsangi	Nsangi Fish Farm	Private
	Matugga	Matugga Fish Farm Limited	Private
	Sissa	Kaama Fish Farm	Private
	Kasanje	Nina Fish Farm	Private
	Kira	Kireka Fish Farm and Hatchery	Private
Mukono	Mukono municipality	Mukono Zonal Aquaculture Research and Development Institute	Public
	Goma	Manjorie Fish Farm	Private
	Mpaata	Lake Victoria Precious Fish Farm Ltd.	Private
Buikwe	Buikwe Rural	Ferdsult Fish Project	Private
	Nyenga	Yalelo fish farm	Private
	Ngogwe	Agrofish Farm	Private
Luwero	Zirobwe	Sanga Fish Farm	Private
Masaka City	Kingo	Ssenya Fish Farm	Private
Kyotera	Kirumba	Pokino Multipurpose Fish Project	Private
Mbarara	Kakiika Division	Mbarara Zonal Aquaculture Research and Development Institute	Public
Rwampara	Bugamba	Dejafa Farm	Private
	Bugamba	Nyakasana Farm	Private
Bushenyi	Central Division	Ruhandagazi Regional Fry Centre	Public
	Kyamuhunga	Kabeihura Farmers	Private
Kanungu	Kihihi Town Council	Kihihi Fish Fry Centre	Public
	Kirima	Waako Fry Centre	Private
Kabale	Ndorwa	Kachwekano Zonal Agricultural Research and Development Institute	Public
Rukungiri	Rukungiri Municipality	Rural Aquaculture Development	Private
Hoima	Kyabigambire	Bulindi Zonal Aquaculture Research and Development Institute	Public
	Kibanjwa	AA Fisheries and Aquaculture Farm	Private
Kasese	Maliba	Nehemia Hatchery	Private
	Kisinga	Blue Valley Fish Farm	Private
Kabarole	Rwengaju Model	Rwebitaba Zonal Agricultural Research and Development Institute	Public
	Mugusu Town council	Adolf Fish Farm	Private
Fort Portal City	Western Division	GEOB Hatchery	Private
Kamwenge	Kahungye Town Council, Rwenkuba	Rubumba Seed Production Research and Training Centre	Private
Mbale city	Northern Division	Mbale Regional Fish Fry Centre	Public
Bushenyi	Kyamuhunga-Mushunga	Kabehura Farm Limited	Private
Busia	Buteba	Salama Integrated Fish Farm Limited	Private
Sironko	Bumalimba	Nalugugu Fish Farm	Private
Bugiri	Buwuni	Kange Integrated Fish Farm	Private
Iganga	Northern Division	Muso4f Enterprises	Private
Mayuge	Wairasa	MIG Fish Farm	Private
Namutumba	Nsinze	Busoga Farmers Resource	Private
Kumi	Atutur	Kumi Wetland Fish Farming Association	Private
Tororo	Western Division	Bamukwasi Rock Valley Fish Farm	Private
	Eastern Division	Rock Springs Fish Farm Limited	Private
	Morukatipe	Geossy Fish Farm and Hatchery	Private
Serere	Olio	Kikoota Integrated Fish Farm	Private
Arua city	Ayivu East Division	Abi Zonal Agricultural Research and Development Institute	Public
Koboko	Nyangilia	Manada Fish Farm	Private
Maracha	Kijomoro	Eyofia Memorial Farm Kochi Ltd.	Private
	Abinyu cell	Wole Mixed Farm	Private
Maracha	Kijomoro	Neville Long Bottom Mixed Farm	Private
Amuru	Pabbo Town Council	Lalar Fish Farm	Private

Disclaimer: To the best of our knowledge, the list of hatcheries presented in this table is correct as of now, but the status of hatcheries and farms changes at least annually.

## Data Availability

Not applicable.

## References

[1] FAO (2022). The State of World Fisheries and Aquaculture 2022. Towards Blue Transformation.

[2] Verdegem M., Buschmann A.H., Latt U.W., Dalsgaard A.J.T., Lovatelli A. (2023). The Contribution of Aquaculture Systems to Global Aquaculture Production. J. World Aquac. Soc..

[3] Naylor R.L., Hardy R.W., Buschmann A.H., Bush S.R., Cao L., Klinger D.H., Little D.C., Lubchenco J., Shumway S.E., Troell M. (2021). A 20-Year Retrospective Review of Global Aquaculture. Nature.

[4] Mapfumo B. (2022). Regional Review on Status and Trends in Aquaculture Development in Sub-Saharan Africa—2020. FAO Fisheries and Aquaculture Circular N. 1232/4.

[5] Zengeya T.A., Booth A.J., Chimimba C.T. (2015). Broad Niche Overlap between Invasive Nile tilapia (*Oreochromis niloticus*, L. 1758) and Indigenous Congenerics in Southern Africa: Should We Be Concerned?. Entropy.

[6] El-Sayed A.F.M., Fitzsimmons K. (2023). From Africa to the World—The Journey of Nile tilapia. Rev. Aquac..

[7] Geletu T.T., Zhao J. (2023). Genetic Resources of Nile tilapia (*Oreochromis niloticus*, L. 1758) in Its Native Range and Aquaculture. Hydrobiologia.

[8] Eknath A.E., Hulata G. (2009). Use and Exchange of Genetic Resources of Nile tilapia (*Oreochromis niloticus*, L. 1758). Rev. Aquac..

[9] Trewavas E. (1983). Tilapiine Fishes of the Genera Sarotherodon, Oreochromis, and Danakilia.

[10] Fitzsimmons K. Supply and Demand in Tilapia Markets and Vietnam’s Role. Proceedings of the Aquaculture 2017 in Can Tho.

[11] Munguti J.M., Nairuti R., Iteba J.O., Obiero K.O., Kyule D., Opiyo M.A., Abwao J., Kirimi J.G., Outa N., Muthoka M. (2022). Nile Tilapia (*Oreochromis niloticus*, L. 1758) Culture in Kenya: Emerging Production Technologies and Socio-Economic Impacts on Local Livelihoods. Aquac. Fish Fish..

[12] Abaho I., Akoll P., Jones C.L.W., Masembe C. (2022). Dietary Inclusion of Pine Pollen Alters Sex Ratio and Promotes Growth of Nile tilapia (*Oreochromis niloticus*, L. 1758). Aquac. Rep..

[13] El-Sayed A.-F.M. (2019). Tilapia Culture.

[14] Prabu E., Rajagopalsamy C.B.T., Ahilan B., Jeevagan I.J.M.A., Renuhadevi M. (2019). Tilapia—An Excellent Candidate Species for World Aquaculture: A Review. Annu. Res. Rev. Biol..

[15] Anane-Taabeah G., Frimpong E.A., Hallerman E. (2019). Aquaculture-Mediated Invasion of the Genetically Improved Farmed Tilapia (Gift) into the Lower Volta Basin of Ghana. Diversity.

[16] Barría A., Peñaloza C., Papadopoulou A., Mahmuddin M., Doeschl-Wilson A., Benzie J.A.H., Houston R.D., Wiener P. (2023). Genetic Differentiation Following Recent Domestication Events: A Study of Farmed Nile tilapia (*Oreochromis niloticus*, L. 1758) Populations. Evol. Appl..

[17] Dey M.M., Eknath A.E., Sifa L., Hussain M.G., Thien T.M., Van Hao N., Aypa S., Pongthana N. (2000). Performance and Nature of Genetically Improved Farmed Tilapia: A Bioeconomic Analysis. Aquac. Econ. Manag..

[18] Abaho I., Masembe C., Akoll P., Jones C.L.W. (2022). The Use of Plant Extracts to Control Tilapia Reproduction: Current Status and Future Perspectives. J. World Aquac. Soc..

[19] Dey M.M. (2000). The Impact of Genetically Improved Farmed Nile tilapia in Asia. Aquac. Econ. Manag..

[20] Abwao J., Jung’a J., Barasa J.E., Kyule D., Opiyo M., Awuor J.F., Ogello E., Munguti J.M., Keya G.A. (2021). Selective Breeding of Nile tilapia (*Oreochromis niloticus*, L. 1758): A Strategy for Increased Genetic Diversity and Sustainable Development of Aquaculture in Kenya. J. Appl. Aquac..

[21] Ansah Y.B., Frimpong E.A., Hallerman E.M. (2014). Genetically-Improved Tilapia Strains in Africa: Potential Benefits and Negative Impacts. Sustainability.

[22] Ragasa C., Charo-Karisa H., Rurangwa E., Tran N., Shikuku K.M. (2022). Sustainable Aquaculture Development in Sub-Saharan Africa. Nat. Food.

[23] FAO (2023). Lessons from Two Decades of Tilapia Genetic Improvement in Africa—Genetics in Aquaculture. A Case Study.

[24] Rezk M.A., Ponzoni R.W., Khaw H.L., Kamel E., Dawood T., John G. (2009). Selective Breeding for Increased Body Weight in a Synthetic Breed of Egyptian Nile tilapia (*Oreochromis niloticus*, L. 1758): Response to Selection and Genetic Parameters. Aquaculture.

[25] Ibrahim N.A., Mohamed Nasr-Allah A., Charo-Karisa H. (2019). Assessment of the Impact of Dissemination of Genetically Improved Abbassa Nile tilapia Strain (GIANT-G9) versus Commercial Strains in Some Egyptian Governorates. Aquac. Res..

[26] Ibrahim N.A., Zaid M.Y.A., Khaw H.L., El-Naggar G.O., Ponzoni R.W. (2013). Relative Performance of Two Nile tilapia (*Oreochromis niloticus*, L. 1758) Strains in Egypt: The Abbassa Selection Line and the Kafr El Sheikh Commercial Strain. Aquac. Res..

[27] Tran N., Shikuku K.M., Rossignoli C.M., Barman B.K., Cheong K.C., Ali M.S., Benzie J.A.H. (2021). Growth, Yield and Profitability of Genetically Improved Farmed Tilapia (GIFT) and Non-GIFT Strains in Bangladesh. Aquaculture.

[28] Hamilton M.G., Lind C.E., Barman B.K., Velasco R.R., Danting M.J.C., Benzie J.A.H. (2020). Distinguishing between Nile tilapia Strains Using a Low-Density Single-Nucleotide Polymorphism Panel. Front. Genet..

[29] Gaikwad A., Padiyar A., Datta S., Shikuku K.M., Mohan V., Trong T., Benzie J., Phillips M. (2021). Dissemination and Scaling Strategy for Genetically Improved Farmed Tilapia (GIFT) in India, 2020–2030. Strategy.

[30] Hinrichsen E., Walakira J.K., Langi S., Ibrahim N.A., Tarus V., Badmus O., Baumüller H. (2022). Prospects for Aquaculture Development in Africa: A Review of Past Performance to Assess Future Potential. Working Paper 211.

[31] Shikuku K.M., Ochenje I., Muthini D. (2021). A Review of the Performance of Fish Seed Systems in Africa. Program Report.

[32] Adeleke B., Robertson-Andersson D., Moodley G., Taylor S. (2021). Aquaculture in Africa: A Comparative Review of Egypt, Nigeria, and Uganda Vis-À-Vis South Africa. Rev. Fish. Sci. Aquac..

[33] Kasozi N., Rutaisire J., Nandi S., Sundaray J.K. (2017). A Review of Uganda and Indias Freshwater Aquaculture: Key Practices and Experience from Each Country. J. Ecol. Nat..

[34] Mwanja M.T., Kityo G., Achieng P., Kasozi J.M., Sserwadda M., Namulawa V.T. (2016). Growth Performance Evaluation of Four Wild Strains and One Current Farmed Strain of Nile tilapia in Uganda. Int. J. Fish. Aquat. Stud..

[35] Aanyu M., Denis O., Cassius A., Gertrude A. (2020). Potential for Enhancing and Sustaining Commercial Aquaculture in Uganda: Producer Organizations, Contract Farming Schemes and Public-Private Partnerships. Int. J. Fish. Aquat. Stud..

[36] Abaho I., Zaabwe T., Izaara A., Kasigwa N.H., Mushabe N., Byenkya S., Nkambo M., Baguma D.S., Hafashimana L.N.D., Efitre J. (2020). Effect of Stocking Density on Growth and Survival of Nile tilapia (*Oreochromis niloticus*, L. 1758) under Cage Culture in Lake Albert, Uganda. Int. J. Fish. Aquac..

[37] FAO Fisheries and Aquaculture—National Aquaculture Sector Overview—Uganda. https://www.fao.org/fishery/en/countrysector/ug/en?lang=en.

[38] MAAIF (2017). National Fisheries and Aquaculture Policy. Ministry of Agriculture, Animal Industry and Fisheries.

[39] Musinguzi L., Lugya J., Rwezawula P., Kamya A., Nuwahereza C., Halafo J., Kamondo S., Njaya F., Aura C., Shoko A.P. (2019). The Extent of Cage Aquaculture, Adherence to Best Practices and Reflections for Sustainable Aquaculture on African Inland Waters. J. Great Lakes Res..

[40] Kwikiriza G., Barekye A., Aheisibwe A., Byakora E. (2017). Comparative Growth Performance and Proximate Nutrient Composition of Three Local Strains of Nile tilapia (*Oreochromis niloticus*, L. 1758) Collected from Different Locations in Uganda. Fish. Aqua. J..

[41] Atukunda G., Atekyereza P., Walakira J., State A.E. (2020). Increasing Farmers’ Access to Aquaculture Extension Services: Lessons from Central and Northern Uganda. Uganda J. Agric. Sci..

[42] Ondhoro C., Kagolola I., Osipa G., Odong R., Kubiriza G., Owere L. (2023). Growth and Economic Evaluation of Different Fish Species for Culture in Uganda’s Mid Altitude Areas Using Local Feeds. J. Appl. Sci..

[43] Walakira J., Akoll P., Engole M., Sserwadda M., Nkambo M., Namulawa V., Kityo G., Musimbi F., Abaho I., Kasigwa H. (2015). Common Fish Diseases and Parasites Affecting Wild and Farmed Tilapia and Catfish in Central and Western Uganda. Uganda J. Agric. Sci..

[44] Abaho I., Gabriel N.N., Izaara A.A., Gabriel N.N., Omoregie E., Abasubong K.P. (2023). Use of Plant Extracts to Control Reproduction in Tilapia Production Systems: An Emerging Eco-Friendly Innovation. Emerging Sustainable Aquaculture Innovations in Africa. Sustainability Sciences in Asia and Africa.

[45] Kwikiriza G., Yegon M.J., Byamugisha N., Beingana A., Atukwatse F., Barekye A., Nattabi J.K., Meimberg H. (2023). Morphometric Variations of Nile tilapia (*Oreochromis niloticus*, L. 1758) Local Strains Collected from Different Fish Farms in South Western Highland Agro-Ecological Zone (SWHAEZ), Uganda: Screening Strains for Aquaculture. Fishes.

[46] Mwanja M., Ondhoro C., Sserwada M., Achieng P., Ddungu R., Mwanja W. (2016). Morphological Variation of Nile tilapia Populations from Major Water Bodies of Uganda. Uganda J. Agric. Sci..

[47] Mwanja M., Rutaisire J., Ondhoro C., Ddungu R., Aruho C. (2015). Current Fish Hatchery Practices in Uganda: The Potential for Future Investment. Int. J. Fish. Aquat. Stud..

[48] Gupta M.V., Acosta B.O. (2004). From Drawing Board to Dining Table: The Success Story of the GIFT Project. NAGA WorldFish Center Quarterly.

[49] Khan S., Hossain M., Hossain M. (2008). Production and Economics of GIFT Strain of Tilapia (*Oreochromis niloticus*, L. 1758) in Small Seasonal Ponds. Progress. Agric..

[50] Das U.N., Jana P., Pahari T., Roy A., Dhara K. (2018). Comparative Study on Growth Performance and Economics between GIFT and Local Varieties of *Oreochromis niloticus* (L. 1758) in Pond Culture Systems. Int. J. Pure Appl. Biosci..

[51] Abwao J., Kyule D., Junga J.O., Barasa J.E., Sigana D.A. (2023). On-farm Growth Performance of Different Strains of tilapia (*Oreochromis niloticus*, L. 1758) Reared in Earthen Ponds. Aquac. Fish Fish..

[52] Dickson M., Nasr-Allah A., Kenawy D., Kruijssen F. (2016). Increasing Fish Farm Profitability through Aquaculture Best Management Practice Training in Egypt. Aquaculture.

[53] FAO Fishery and Aquaculture Statistics. Global Aquaculture Production 1950–2021 (FishStatJ)..

[54] Ambekar E., Madan M.D., Morten R., Bjarne G., Abella T.A., Ruben C.S., Tayamen M.M., Reyes R.A., Hans B.B. Selective Breeding of Nile Tilapia for Asia. Proceedings of the 6th World Congress of Genetics Applied to Livestock Production.

[55] Ponzoni R.W., Nguyen N.H., Khaw H.L., Hamzah A., Bakar K.R.A., Yee H.Y. (2011). Genetic Improvement of Nile tilapia (*Oreochromis niloticus*, L. 1758) with Special Reference to the Work Conducted by the World Fish Center with the GIFT Strain. Rev. Aquac..

[56] Yáñez J.M., Joshi R., Yoshida G.M. (2020). Genomics to Accelerate Genetic Improvement in Tilapia. Anim. Genet..

[57] Mwanja M.T., Mwanja W.W. (2009). Escape of Farmed Tilapiines into the Wild and Entry of Wild Forms in Fishponds, and the Possible Interactions between Wild and Farmed Tilapiines from a Sample of Smallholder Farms in Central Uganda. Afr. J. Ecol..

[58] Gjedrem T., Robinson N., Rye M. (2012). The Importance of Selective Breeding in Aquaculture to Meet Future Demands for Animal Protein: A Review. Aquaculture.

[59] Liu S., Wang C., Li C. (2018). Progress in Aquaculture Genetics and Breeding in China. J. World Aquac. Soc..

[60] Kang X., Wei D., Jun X., Min T., Chun Z., Yun L., ShaoJun L. (2015). Development and Application of Biological Technologies in Fish Genetic Breeding. Sci. China Life Sci..

[61] Brummett R.E., Ponzoni R.W. (2009). Concepts, Alternatives, and Environmental Considerations in the Development and Use of Improved Strains of Tilapia in African Aquaculture. Rev. Fish. Sci..

[62] Gjedrem T., Robinson N. (2014). Advances by Selective Breeding for Aquatic Species: A Review. Agric. Sci..

[63] Thodesen J., Rye M., Wang Y.X., Yang K.S., Bentsen H.B., Gjedrem T. (2011). Genetic Improvement of Tilapias in China: Genetic Parameters and Selection Responses in Growth of Nile tilapia (*Oreochromis niloticus*, L. 1758) after Six Generations of Multi-Trait Selection for Growth and Fillet Yield. Aquaculture.

[64] Symonds J.E., Clarke S.M., King N., Walker S.P., Blanchard B., Sutherland D., Roberts R., Preece M.A., Tate M., Buxton P. (2019). Developing Successful Breeding Programs for New Zealand Aquaculture: A Perspective on Progress and Future Genomic Opportunities. Front. Genet..

[65] Gjedrem T. (2012). Genetic Improvement for the Development of Efficient Global Aquaculture: A Personal Opinion Review. Aquaculture.

[66] Rye M. Current Status and Prospects for the Application of Genetic Improvement in Aquaculture Species. Proceedings of the 9th Biennial Symposium of the Brazilian Society of Animal Breeding.

[67] Komen H., Trong T.Q. Nile Tilapia Genetic Improvement: Achievements and Future. Proceedings of the 10th International Symposium on Tilapia in Aquaculture—ISTA10.

[68] Neira R. Breeding in Aquaculture Species: Genetic Improvement Programs in Developing Countries. Proceedings of the 9th World Congress on Genetics Applied to Livestock Production.

[69] Gjedrem T. (2010). The First Family-Based Breeding Program in Aquaculture. Rev. Aquac..

[70] Rye M., Gjerde B., Gjedrem T. Genetic Improvement Programs for Aquaculture Species in Developed Countries. Proceedings of the 9th World Congress on Genetics Applied to Livestock Production.

[71] Ponzoni R.W., Nguyen N.H., Khaw H.L. (2007). Investment Appraisal of Genetic Improvement Programs in Nile tilapia (*Oreochromis niloticus*, L. 1758). Aquaculture.

[72] Lind C.E., Ponzoni R.W., Nguyen N.H., Khaw H.L. (2012). Selective Breeding in Fish and Conservation of Genetic Resources for Aquaculture. Reprod. Domest. Anim..

[73] Ponzoni R.W., Nguyen N.H., Khaw H.L., Ninh N.H. (2008). Accounting for Genotype by Environment Interaction in Economic Appraisal of Genetic Improvement Programs in Common carp (*Cyprinus carpio*). Aquaculture.

[74] Eze F. (2019). Marker-Assisted Selection in Fish: A Review. Asian J. Fish. Aquat..

[75] Abdelrahman H., ElHady M., Alcivar-Warren A., Allen S., Al-Tobasei R., Bao L., Beck B., Blackburn H., Bosworth B., Buchanan J. (2017). Aquaculture Genomics, Genetics and Breeding in the United States: Current Status, Challenges, and Priorities for Future Research. BMC Genom..

[76] Song H., Dong T., Yan X., Wang W., Tian Z., Sun A., Dong Y., Zhu H., Hu H. (2022). Genomic Selection and Its Research Progress in Aquaculture Breeding. Rev. Aquac..

[77] Houston R.D., Bean T.P., Macqueen D.J., Gundappa M.K., Jin Y.H., Jenkins T.L., Selly S.L.C., Martin S.A.M., Stevens J.R., Santos E.M. (2020). Harnessing Genomics to Fast-Track Genetic Improvement in Aquaculture. Nat. Rev. Genet..

[78] Olesen I., Gjedrem T., Bentsen H.B., Gjerde B., Rye M. (2003). Breeding Programs for Sustainable Aquaculture. J. Appl. Aquac..

[79] Nguyen N.H., Whatmore P., Miller A., Knibb W. (2016). Quantitative Genetic Properties of Four Measures of Deformity in Yellowtail Kingfish (*Seriola lalandi*, V. 1833). J. Fish. Dis..

[80] Ponzoni R.W., Acosta B.O., Ponniah A.G. (2006). Development of Aquatic Animal Genetic Improvement and Dissemination Programs: Current Status and Action Plans.

[81] Gjedrem T., Rye M. (2018). Selection Response in Fish and Shellfish: A Review. Rev. Aquac..

[82] Teletchea F. (2021). Fish Domestication in Aquaculture: 10 Unanswered Questions. Anim. Front..

[83] Kristjánsson Ó.H., Gjerde B., Ødegård J., Lillehammer M. (2020). Quantitative Genetics of Growth Rate and Filet Quality Traits in Atlantic Salmon Inferred from a Longitudinal Bayesian Model for the Left-Censored Gaussian Trait Growth Rate. Front. Genet..

[84] Bentsen H.B., Gjerde B., Eknath A.E., de Vera M.S.P., Velasco R.R., Danting J.C., Dionisio E.E., Longalong F.M., Reyes R.A., Abella T.A. (2017). Genetic Improvement of Farmed Tilapias: Response to Five Generations of Selection for Increased Body Weight at Harvest in *Oreochromis niloticus* (L. 1758) and the Further Impact of the Project. Aquaculture.

[85] Ponzoni R.W., Nguyen N.H., Khaw H.L., Rodriguez B.M. (2012). Considerations about Effective Dissemination of Improved Strains. Working Paper 2012-47.

[86] Tave D. (1999). Inbreeding and Broodstock Management. Fisheries Technical Paper. No. 392.

[87] Kajungiro R.A., Mapenzi L.L., Nyinondi C.S., Haldén A.N., Mmochi A.J., Chacha M., Mtolera M.S., Lamtane H.A., Jan De Koning D. (2019). The Need of a Structured Tilapia Breeding Program in Tanzania to Enhance Aquaculture Production: A Review. Tanzan J. Sci..

[88] Bentsen H.B., Olesen I. (2002). Designing Aquaculture Mass Selection Programs to Avoid High Inbreeding Rates. Aquaculture.

[89] Bolivar R.B., Sayco V.M.R., Jimenez T.E.B., Argueza B.R.L., Bolivar H.L., Dadag L.B., Taduan A.G., Borski R.J. (2009). Broodstock Seed Quality and Fingerling Production Systems Rearing for Nile Tilapia in the Philippines. Technical Report.

[90] Ingthamjitr S., Paankhao N., Paankhao S., Promsri K. (2017). Effect of Maternal Age on Reproductive Reproductive Performance and Growth of Nile tilapia (*Oreochromis niloticus*, L. 1758) Fry. J. Fish. Environ..

[91] Thodesen J., Rye M., Lozano C., Avitua V.S., Johansen H., Segovia H., Ospina J. (2016). Selective Breeding of Tilapia: Status and Prospects.

[92] Ponzoni R.W., Khaw L., Yee H.Y. (2010). GIFT: The Story Since Leaving ICLARM (Now Known as The WorldFish Center). Socioeconomic, Access and Benefit Sharing and Dissemination Aspects. FNI Report 14/2010.

[93] Eknath A.E., Tayamen M.M., Palada-de Vera M.S., Danting J.C., Reyes R.A., Dionisio E.E., Capili J.B., Bolivar H.L., Abella T.A., Circa A.V. (1993). Genetic Improvement of Farmed Tilapias: The Growth Performance of Eight Strains of *Oreochromis niloticus* (L. 1758) Tested in Different Farm Environments. Genetics in Aquaculture.

[94] Pulin R.S.V., Eknath A.E., Gjedrem T., Tayamen M.M., Macaranas J.E., Abella T.A. (1991). The Genetic Improvement of Farmed Tilapias (GIFT) Project: The Story So Far. NAGA ICLARM] Quarterly; Contributions No 721.

[95] Sanda M.K., Metcalfe N.B., Mable B.K. (2024). The Potential Impact of Aquaculture on the Genetic Diversity and Conservation of Wild Fish in Sub-Saharan Africa. Aquat. Conserv. Mar. Freshw. Ecosyst..

[96] Hamzah A., Ponzoni R.W., Nguyen N.H., Khaw H.L., Yee H.Y., Nor S.A.M. (2014). Performance of the Genetically Improved Farmed Tilapia (GIFT) Strain over Ten Generations of Selection in Malaysia. Pertanika J. Trop Agric. Sci..

[97] Ordoñez J.F.F., Santos M.D., Tayamen M.M. (2014). Tilapia Genetic R&D in the Philippines: Challenges and Prospects for Future Development.

[98] Eknath A.E., Acosta B.O. (1998). Genetic Improvement of Farmed Tilapias (GIFT) Project: Final Report, March 1988 to December 1997.

[99] Eknath A.E., Bentsen H.B., Ponzoni R.W., Rye M., Nguyen N.H., Thodesen J., Gjerde B. (2007). Genetic Improvement of Farmed Tilapias: Composition and Genetic Parameters of a Synthetic Base Population of *Oreochromis niloticus* (L. 1758) for Selective Breeding. Aquaculture.

[100] Acosta B.O., Sevilleja R.C., Gupta M.V. (2006). Public and Private Partnerships in Aquaculture. A Case Study on Tilapia Research and Development.

[101] Bolivar R.B., Newkirk G.F. (2002). Response to within Family Selection for Body Weight in Nile tilapia (*Oreochromis niloticus*, L. 1758) Using a Single-Trait Animal Model. Aquaculture.

[102] Bolivar R.B. (1998). Estimation of Response to Within-Family Selection for Growth in Nile tilapia (*Oreochromis niloticus*, L. 1758). Ph.D. Thesis.

[103] El-Sayed A.M. (2006). Tilapia Culture.

[104] Tayamen M.M. Nationwide Dissemination of GET EXCEL Tilapia in the Philippines. Proceedings of the 6th International Symposium on Tilapia in Aquaculture.

[105] ADB (2005). An Impact Evaluation of the Development of Genetically Improved Farmed Tilapia and Their Dissemination in Selected Countries.

[106] FAO (2018). Aquaculture Development. Development of Aquatic Genetic Resources. A Framework of Essential Criteria. TG5 Suppl. 9.

[107] Li S.-F., Cai W.-Q. Contribution of Genetic Improved Strains to Chinese Tilapia Industry. Proceedings of the 8th International Symposium on Tilapia in Aquaculture.

[108] Dey M.M., Gupta M.V. (2000). Socioeconomics of Disseminating Genetically Improved Nile tilapia in Asia: An Introduction. Aquac. Econ. Manag..

[109] Li L., Dong Z., Su S., Xu P., Liang Z., Ma L., Liu W., Zhang J. (2012). Morphological Variation and Mathematical Analysis of Effects of Morphological Traits on Body-Weight of GIFT Tilapia after 3 Generations of Breeding. J. Fish. China.

[110] Said M. (2016). Reproductive Performance and Early Growth of Three Strains of Nile tilapia (*Oreochromis niloticus*, L. 1758) in Egypt: Abbassa, Kafr El Sheikh, and Manzala. J. Anim. Poul. Fish. Prod..

[111] Trinh T.Q., Agyakwah S.K., Khaw H.L., Benzie J.A.H., Attipoe F.K.Y. (2021). Performance Evaluation of Nile tilapia (*Oreochromis niloticus*, L. 1758) Improved Strains in Ghana. Aquaculture.

[112] Hussain M.G. (2009). A Future for the Tilapia in Bangladesh. AQUA Culture Industry Review.

[113] Luan T.D., Thien T.M., Luu L.T., Hoa N.T. (2007). Breeding Programme and Nationwide Dissemination of NOVIT 4 Tilapia in Vietnam. Proceedings of the 25th Anniversary Scientific Conference of NARA on Tropical Aquatic Research towards Sustainable Development, 2007, Research Institute for Aquaculture No.1 (RIA.1).

[114] Nguyen N.H. (2016). Genetic Improvement for Important Farmed Aquaculture Species with a Reference to Carp, Tilapia and Prawns in Asia: Achievements, Lessons, and Challenges. Fish Fish..

[115] Nguyen H.N., Ponzoni R.W., Chandrasoma J., Herath H., Wathurawadu K. (2011). GIFT Tilapia Raise Culture Efficiency in Sri Lanka.Global Aquaculture Advocate.

[116] Yoshida G.M., de Oliveira C.A.L., Campos E.C., Todesco H., Araújo F.C.T., Karin H.M., Zardin A.M.S.O., Bezerra Júnior J.S., Filho L.A., Vargas L. (2022). A Breeding Program for Nile Tilapia in Brazil: Results from Nine Generations of Selection to Increase the Growth Rate in Cages. J. Anim. Breed. Genet..

[117] De Oliveira C.A.L., Ribeiro R.P., Yoshida G.M., Kunita N.M., Rizzato G.S., de Oliveira S.N., dos Santos A.I., Nguyen N.H. (2016). Correlated Changes in Body Shape after Five Generations of Selection to Improve Growth Rate in a Breeding Program for Nile tilapia (*Oreochromis niloticus*, L. 1758) in Brazil. J. Appl. Genet..

[118] Aquabel A Premium Tilapia Brand with 25 Years of Product Development in Brazil. https://genomar.com/products-brands/.

[119] Almeida D.B., da Costa M.A.P., Bassini L.N., Calabuig C.I.P., Moreira C.G.A., Rodrigues M.D.N., Pérez H.J., Tavares R.A., Varela A.S., Moreira H.L.M. (2013). Reproductive Performance in Female Strains of Nile tilapia (*Oreochromis niloticus*, L. 1758). Aquac. Int..

[120] Carvalhoa J.C., Filhoa C.R.A.C., Oliveirab C.A.L., Ribeirob R.P., Seraphima G.N., Silvaa A.L.N., Kinjo Juniora G.N., Laicec L.M., Fantinid L.E., Lopera-Barreroe N.M. (2022). Growth Curve of Nile tilapia from Different Families of the AquaAmérica Variety. Braz. J. Biol..

[121] Ataguba G.A., Ikongbeh A.O., Garba A.A. (2020). Reciprocal Crosses of Two Improved Strains of Nile tilapia: Implications on Reproductive and Growth Traits. J. Res. Agric. Anim. Sci..

[122] Sukmanomon S., Kamonrat W., Poompuang S., Nguyen T.T.T., Bartley D.M., May B., Na-Nakorn U. (2012). Genetic Changes, Intra- and Inter-Specific Introgression in Farmed Nile tilapia (*Oreochromis niloticus*, L. 1758) in Thailand. Aquaculture.

[123] Tibihika P.D., Meimberg H., Curto M. (2022). Understanding the Translocation Dynamics of Nile tilapia (*Oreochromis niloticus*, L. 1758) and Its Ecological Consequences in East Africa. Afr. Zool..

[124] Bradbeer S.J., Harrington J., Watson H., Warraich A., Shechonge A., Smith A., Tamatamah R., Ngatunga B.P., Turner G.F., Genner M.J. (2019). Limited Hybridization between Introduced and Critically Endangered Indigenous Tilapia Fishes in Northern Tanzania. Hydrobiologia.

[125] Deines A.M., Bbole I., Katongo C., Feder J.L., Lodge D.M. (2014). Hybridisation between Native *Oreochromis* Species and Introduced Nile tilapia (*Oreochromis niloticus*, L. 1758) in the Kafue River, Zambia. Afr. J. Aquat. Sci..

[126] Kariuki J., Tibihika P.D., Curto M., Alemayehu E., Winkler G., Meimberg H. (2021). Application of Microsatellite Genotyping by Amplicon Sequencing for Delimitation of African Tilapiine Species Relevant for Aquaculture. Aquaculture.

[127] Muhlfeld C.C., Kalinowski S.T., McMahon T.E., Taper M.L., Painter S., Leary R.F., Allendorf F.W. (2009). Hybridization Rapidly Reduces Fitness of a Native Trout in the Wild. Biol. Lett..

[128] Tibihika P.D., Curto M., Alemayehu E., Waidbacher H., Masembe C., Akoll P., Meimberg H. (2020). Molecular Genetic Diversity and Differentiation of Nile tilapia (*Oreochromis niloticus*, L. 1758) in East African Natural and Stocked Populations. BMC Evol. Biol..

[129] Robledo D., Ogwang J., Byakora E., Schulze J.N., Benda K.K., Fraslin C., Salisbury S., Solimo M., Mayega J.F., Peter B. (2024). Genetic Diversity and Population Structure of Farmed and Wild Nile tilapia (*Oreochromis niloticus*, L. 1758) in Uganda: The Potential for Aquaculture Selection and Breeding Programs. Genomics.

[130] Bolman B., Van Duijn A.P., Rutaisire J. (2018). Review and Analysis of Small-Scale Aquaculture Production in East Africa. Part 4 Uganda, Report WCDI-18-021.

[131] Fessehaye Y., Komen H., Rezk M.A., van Arendonk J.A.M., Bovenhuis H. (2007). Effects of Inbreeding on Survival, Body Weight and Fluctuating Asymmetry (FA) in Nile tilapia (*Oreochromis niloticus*, L. 1758). Aquaculture.

[132] Khan M.G.Q. (2011). Marker-Assisted Selection in Enhancing Genetically Male Nile tilapia (*Oreochromis niloticus*, L. 1758) Production. Ph.D. Thesis.

[133] Tibihika P.D., Aruho C., Namulawa V., Ddungu R., Atukunda G., Aanyu M., Nkambo M., Vijayan T., Kwikiriza G., Curto M. (2024). Unlocking Nile tilapia (*Oreochromis niloticus*, L. 1758) Selective Breeding Programmes in Uganda through Geographical Genetic Structure Mapping. Aquac. Fish Fish..

[134] MAAIF (2018). Ministry of Agriculture, Animal Industry and Fisheries Performance Report. Financial Year 2017/2018.

[135] Sae-Lim P., Kause A., Mulder H.A., Martin K.E., Barfoot A.J., Parsons J.E., Davidson J., Rexroad C.E., Van Arendonk J.A.M., Komen H. (2013). Genotype-by-Environment Interaction of Growth Traits in Rainbow Trout (*Oncorhynchus mykiss*): A Continental Scale Study. J. Anim. Sci..

[136] Nguyen N.H., Hamzah A., Thoa N.P. (2017). Effects of Genotype by Environment Interaction on Genetic Gain and Genetic Parameter Estimates in Red tilapia (*Oreochromis* Spp.). Front. Genet..

[137] MAAIF (2012). Department of Fisheries Resource (DiFR) Annual Report 2010/2011.

[138] MAAIF (2003). The Fish (Aquaculture) Rules, 2003. Statutory Instruments 2003 No.81.

[139] MAAIF (2022). The Fish (Aquaculture) Rules, 2022. Statutory Instruments 2022 No.97.

[140] MAAIF (2022). The Fisheries and Aquaculture Act, 2022.

[141] MAAIF (2001). The Animal Breeding Act, 2001.

[142] Janssen K., Saatkamp H., Komen H. (2018). Cost-Benefit Analysis of Aquaculture Breeding Programs. Genet. Sel. Evol..

[143] Fernandes A.F.A., Alvarenga É.R., Alves G.F.O., Manduca L.G., Toral F.L.B., Valente B.D., Silva M.A., Rosa G.J.M., Turra E.M. (2019). Genotype by Environment Interaction across Time for Nile tilapia, from Juvenile to Finishing Stages, Reared in Different Production Systems. Aquaculture.

[144] De Araújo F.C.T., de Oliveira A.L.C., Campos C.E., Yoshida M.G., Lewandowski V., Todesco H., Nguyen H.N., Ribeiro P.R. (2020). Effects of Genotype × Environment Interaction on the Estimation of Genetic Parameters and Gains in Nile tilapia. J. Appl. Genet..

[145] Abd El-Hack M.E., El-Saadony M.T., Nader M.M., Salem H.M., El-Tahan A.M., Soliman M., Khafaga A.F. (2022). Effect of Environmental Factors on Growth Performance of Nile tilapia (*Oreochromis niloticus*, L. 1758). Int. J. Biometeorol..

